# Congenital hemangiomas with transient neonatal thrombocytopenia: case reports and review of the literature

**DOI:** 10.3389/fped.2026.1728203

**Published:** 2026-05-20

**Authors:** Yuan-yang Zheng, Jiajie Lv, Xiaoxi Lin, Chen Hua

**Affiliations:** Department of Plastic and Reconstructive Surgery, Division of Vascular Anomalies, Shanghai Ninth People's Hospital, Shanghai Jiaotong University School of Medicine, Shanghai, China

**Keywords:** congenital hemangioma, kaposiform haemangioendothelioma, KMP, pediatric dermatology, vascular tumors

## Abstract

Congenital hemangioma with thrombocytopenia is rare and is often mistaken for Kasabach-Merritt phenomenon seen in kaposiform hemangioendothelioma. We present three neonatal cases with severe but transient thrombocytopenia that resolved without intervention. Accurate diagnosis, cardiac evaluation, and cautious management are essential, as most patients recover spontaneously, avoiding unnecessary interventions.

## Introduction

Congenital hemangiomas (CH) are fully formed vascular tumors present at birth. Most CHs follow a benign course, but in rare cases, they may be associated with thrombocytopenia. Thrombocytopenia is generally defined as a platelet count <150 × 10^9^/L, with severe thrombocytopenia typically referring to platelet counts <50 × 10^9^/L, particularly in the neonatal period. This phenomenon remains poorly understood and has often been misclassified as Kasabach-Merritt phenomenon (KMP), a distinct coagulopathy typically seen in kaposiform hemangioendothelioma (KHE) or tufted angioma (1). While KMP requires urgent and aggressive treatment, CH-associated thrombocytopenia appears to follow a milder, self-limiting course. Here, we report three cases of full-term neonates with large CHs and early-onset severe thrombocytopenia. We also present the most comprehensive literature review to date, identifying 29 reported patients with CH-associated thrombocytopenia—more than double the 14 cases documented in the largest previous review, thereby emphasizing the importance of accurate diagnosis, conservative management, and differentiation from KMP.

## Case presentation

**Figure 1:**
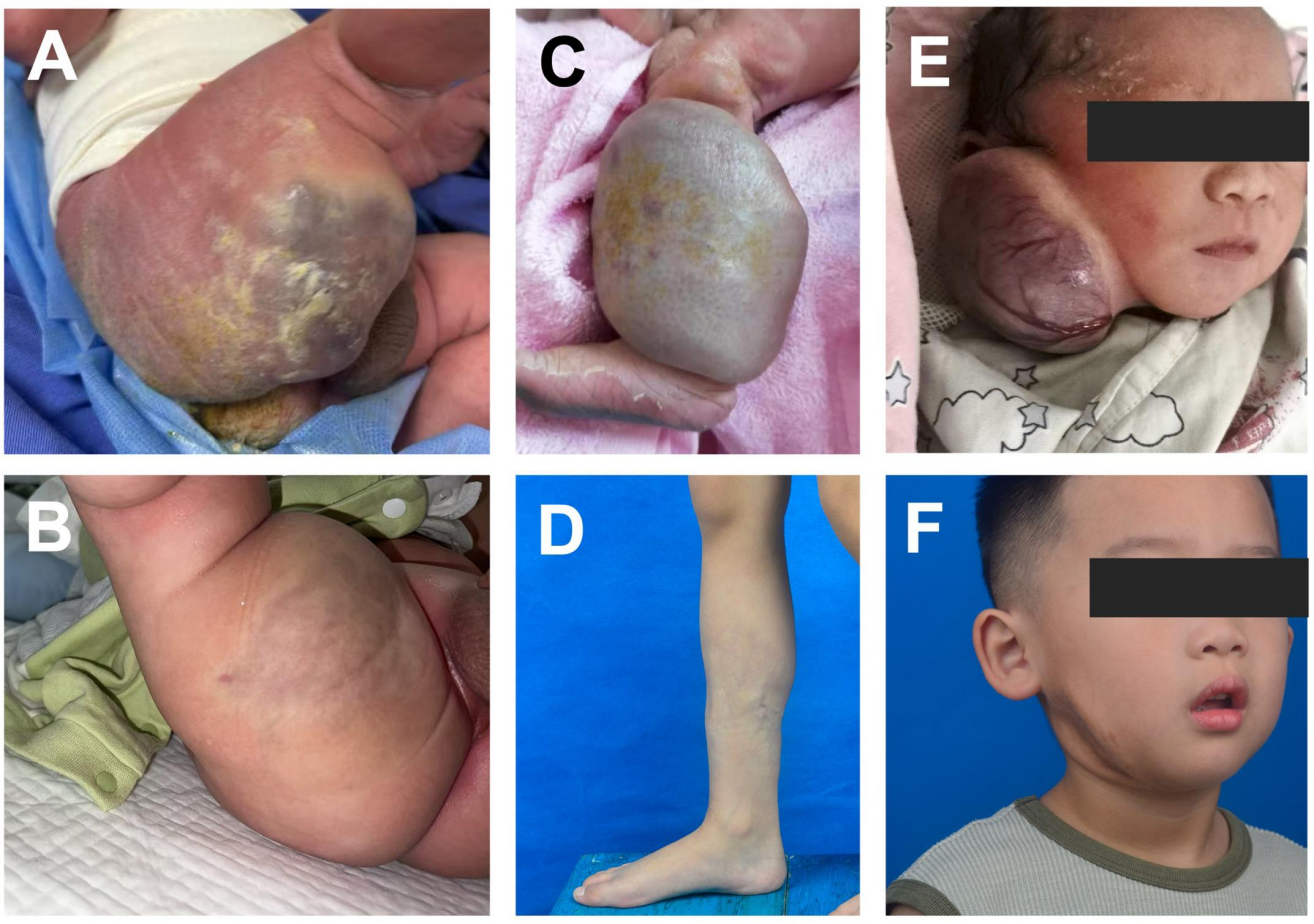
Clinical course of three patients with congenital hemangioma and thrombocytopenia. **(A,B)** Vascular mass on the right thigh of Patient 1 at birth **(A)** and at 3 months of age **(B)**. **(C,D)** Vascular mass on the right lower leg of Patient 2 at birth **(C)** and at 6 years of age **(D)**. **(E,F)** Vascular mass on the right face of Patient 3 at birth **(E)** and at 4 years of age **(F)**.

The first case involved a full-term male infant born via spontaneous vaginal delivery. At birth, a large purple vascular tumor (6 cm × 5 cm) was present on the right buttock and thigh (Figure 1A). The lesion was soft, pulsatile, and warm, with a pale peripheral halo. Thrombocytopenia was detected at birth (platelet count 14 × 10^9^/L), reaching a nadir of 8 × 10^9^/L on day 4. Coagulation studies were within normal limits, with no evidence of consumptive coagulopathy. No clinical bleeding, intracranial hemorrhage, or signs of cardiac dysfunction were observed. Based on a provisional diagnosis of KHE with KMP, sirolimus therapy was initiated on day 3 at the referring hospital. The patient was transferred to our institution where imaging and pathology examination confirmed a final diagnosis of CH, sirolimus was thus discontinued after two months of therapy. Notably, platelet counts recovered spontaneously by day 10, and the lesion showed marked involution by three months (Figure 1B).

The second case was a female infant born at 40 + 5 weeks of gestation via spontaneous vaginal delivery, with a birth weight of 3,150 g. At birth, a bluish-gray, exophytic vascular mass (8 cm × 6 cm) covered most of the right lower leg (Figure 1C). Thrombocytopenia was detected at 10 days of age, with a nadir of 3.8 × 10^9^/L, recovering without intervention. Laboratory evaluation revealed isolated thrombocytopenia with normal coagulation parameters. No bleeding complications or cardiac abnormalities were noted. Based on an initial diagnosis of KMP, sirolimus was administered from 2 to 8 months of age at the referring hospital. However, subsequent pathology and imaging studies at our institution supported a diagnosis of CH. By 20 months, the lesion had significantly regressed, and complete resolution was observed at age six (Figure 1D).

A third case involved a male infant in whom a large facial vascular tumor was detected *in utero* at 32 weeks' gestation. He was delivered by cesarean section at 37 weeks. At birth, a bluish-purple mass (7.2 cm × 7.0 cm × 3.6 cm) involved the right face; the lesion was soft, warm, and encircled by a pale peripheral halo (Figure 1E). Platelet counts remained ∼120 × 10^9^/L during the early neonatal period. On day 32, rupture of a superficial vessel caused external bleeding, requiring emergent vascular ligation; single transfusions of packed red blood cells, plasma, and platelets were administered the same day. Platelet counts then declined, reaching a nadir on day 42, and subsequently recovered, normalizing within 12 days. Apart from transient thrombocytopenia following hemorrhage, coagulation parameters remained within normal limits. No intracranial hemorrhage or cardiac dysfunction was detected during the clinical course. The lesion continued to involute completely. At 4 years of follow-up, the lesion had resolved with residual contour depression (Figure 1F), and ultrasonography showed no appreciable blood flow.

## Discussion

CH associated with thrombocytopenia are exceedingly rare. Most cases involve large vascular tumors, with a mean diameter of 8.8 cm (greatest dimension), compared to 6.0 cm in RICH (2). Interestingly, while non-involuting congenital hemangiomas (NICH) can reach similar sizes, no cases of thrombocytopenia have been reported in NICH. Additionally, while RICH (Rapidly Involuting Congenital Hemangiomas) lesions are most commonly found in the head and neck region, those associated with thrombocytopenia are more likely to occur on the lower limbs (Table 1).

**Table 1:** Summary of published cases of congenital hemangiomas with thrombocytopenia.

Patient	Sex	GA/BW (weeks/g)	Lesion location	Lesion size (cm)	Lowest platelet count (×10^9^/L)/Day	Cardiac failure	Reference
Case 1	M	NA/NA	thigh	6 × 6	NA/NA	-	Boon et al. (2)
Case 2	NA	NA/NA	sacrococcygeal	6 × 6	NA/NA	+	
Case 3	M	40/3,455	thorax	12 × 7.4 × 1.5	54/Day 3	+	Hosono et al. (6)
Case 4	F	36/2,316	scalp	7 × 6 × 3.5	107/Day 3	+	Hsiao et al. (10)
Case 5	M	38/4,000	scalp	6 × 7 × 1.5	56/Day 5	-	Baselga et al. (1)
Case 6	F	41/4,170	arm	8 × 8	62/Day 6	-	
Case 7	M	full-term/NA	leg	NA	5/Day 2	-	
Case 8	M	NA/NA	thorax	8 × 5 × 2.5	7/Day 1	+	
Case 9	M	NA/NA	thigh	5.1 × 4.1	19/Day 2	-	
Case 10	F	NA/NA	arm	10 × 8	21/Day 8	-	
Case 11	M	NA/NA	leg	11 × 13	30/Day 12	-	
Case 12	F	NA/NA	arm	7.5 × 9.3	3/Day 14	-	Rangwala et al. (11)
Case 13	F	38/NA	neck	10 × 14	121/NA	+	Weitz et al. (9)
Case 14	M	full-term/NA	arm	6 × 6	34/Day 16	-	Andreu-Barasoain et al. (12)
Case 15	NA	NA/NA	NA	NA	102/Day 1	-	Braun et al. (13)
Case 16	NA	NA	NA	NA	118/NA	-	
Case 17	NA	NA	NA	NA	100–150/NA	NA	El Zein et al. (14)
Case 18	NA	NA	NA	NA	100–150/NA	NA	
Case 19	NA	NA	NA	NA	100–150/NA	NA	
Case 20	NA	NA	NA	NA	100–150/NA	NA	
Case 21	NA	NA	NA	NA	60/NA	NA	
Case 22	F	32/1,955	thigh	15	55/NA	+	Forjaco Jorge et al. (15)
Case 23	F	36/2,630	maxillofacial region	7 × 7 × 3	19/Day 29	+	Ren et al. (8)
Case 24	M	40/4,515	neck	11 × 10	13/NA	+	Fantasia et al. (7)
Case 25	M	full-term/4,515	neck	15 × 12	13/NA	+	Diociaiuti et al. (16)
Case 26	M	full-term/NA	thigh	10 × 9 × 5	60/Day 4	-	Palma et al. (17)
Case 27	M	full-term/NA	thigh	6 × 5	8/Day 4	-	Zheng et al.
Case 28	F	40^+5^/3,150	leg	8 × 6	3.8/Day 10	-	
Case 29	M	37/2,400	maxillofacial region	7.2 × 7 × 3.6	20/Day 42	-	

GA, gestation age; BW, birth weight.

When encountering a neonate with a vascular tumor and thrombocytopenia, the priority is an accurate diagnosis—particularly distinguishing CH from KHE. Two of our patients were initially misclassified as KHE with KMP at other institutions and received short-term sirolimus unnecessarily, carrying the risk of adverse effects of oral aphtha, herpesvirus reactivation, upper respiratory tract infections and elevation of transaminases (3). Despite overlapping appearances, several features aid in differentiation. First, CH typically presents with a peripheral halo and a soft texture, whereas enlarging KHE lesions are firm with ill-defined borders. Second, ultrasonography and MRI provide distinct imaging features between the two entities. Infiltration and lymphatic component are characteristic of KHE, compared with RICH which is typically well-defined (4). Third, KHE may be complicated by KMP, characterized by profound thrombocytopenia (platelet counts usually <50 × 10^9^/L) and severe coagulopathy, a life-threatening complication requiring urgent intervention (1). By contrast, thrombocytopenia in CH is generally isolated, reaching a nadir in the first week of life, with no significant coagulation abnormalities, and platelet counts recover spontaneously alongside tumor regression. KMP, in our view, is not the appropriate term to describe the abnormalities observed in CH, given that these entities are now confirmed to be biologically distinct from KHE. Ultimately, when clinical differentiation is challenging, histopathology provides the most reliable means of establishing the diagnosis.

Concomitance of thrombocytopenia and high-output heart failure is a severe complication. As shown in Table 1, 9 of 24 cases of CH with thrombocytopenia had heart failure. Concurrent thrombocytopenia and heart failure in CH may be associated with larger lesion (10.7 cm vs. 8.8 cm, Table 1). Therefore, once the diagnosis is established, careful assessment of cardiac function and hemodynamics is essential. Notably, heart failure secondary to KHE complicated by KMP is exceptionally rare, even in cases with extensive lesions; only one such case was reported (5). This finding indicates distinct pathogenic and hemodynamic profiles of the two conditions, providing novel clues for their early differential diagnosis. Management decisions should be dictated by clinical parameters, laboratory results, and hemodynamic stability. In patients with heart failure, tumor embolization or surgical resection has proven effective in stabilizing hemodynamics, whereas transfusion of whole blood or platelets not only offers limited efficacy for thrombocytopenia but may also exacerbate heart failure due to volume overload (2, 6–9). For infants presenting with thrombocytopenia, stable hemodynamics and preserved cardiac function, expectant management with close clinical and laboratory surveillance is appropriate; while medication may not be invariably necessary (1).

In conclusion, CH with thrombocytopenia represent a rare but distinct clinical entity. Accurate diagnosis is essential to avoid unnecessary interventions and to distinguish CH from KHE complicated by KMP. Our cases, together with previous reports, highlight that most CH-associated thrombocytopenia is transient and self-limiting, underscoring the importance of close monitoring and judicious clinical decision-making in neonatal vascular tumors.

## Data Availability

The original contributions presented in the study are included in the article/Supplementary Material, further inquiries can be directed to the corresponding author.
